# Ambient Cold Exposure Modulates B-Cell Distribution via Glucocorticoid-CXCR4 Signaling

**DOI:** 10.3390/ijms27188214

**Published:** 2026-09-15

**Authors:** Hsuan-Yun Lee, Te-Sheng Lien, Der-Shan Sun, Guan-Ling Lin, Ching-Feng Cheng, Ko-Nien Shih, Hsin-Hou Chang

**Affiliations:** 1 Department of Molecular Biology and Human Genetics, Tzu-Chi University, Hualien 970, Taiwan; hsuanyun0915@gmail.com (H.-Y.L.); alan211@gms.tcu.edu.tw (T.-S.L.); dssun@gms.tcu.edu.tw (D.-S.S.); guan0223@gmail.com (G.-L.L.); 2 Department of Pediatrics, Taipei Tzu Chi Hospital, Buddhist Tzu Chi Medical Foundation, New Taipei City 231, Taiwan; chengcf@gms.tcu.edu.tw; 3 Institute of Biomedical Sciences, Academia Sinica, Taipei 115, Taiwan; 4 Department of Medical Image and Radiological Technology, Yuanpei University of Medical Technology, Hsinchu City 300, Taiwan; shihkn@mail.ypu.edu.tw

**Keywords:** B-cell trafficking, cold stress, bone marrow homing, CXCR4, corticosterone, recompartmentalization

## Abstract

Immune cell trafficking among blood, lymphoid organs, and tissues is essential for homeostasis; however, the influence of environmental stressors on steady-state distribution remains unclear. Here we show that ambient cold stress (4 °C) and dexamethasone treatment induced systemic leukocytopenia, marked by the selective depletion of circulating B cells and reduced splenic mass. Viability assays confirmed that this loss was not due to cell death. Adoptive transfer of fluorescently labeled leukocytes revealed the directed redistribution of B cells into the bone marrow (BM). Regarding the mechanism, cold stress elevated corticosterone, which upregulated the homing receptor C-X-C chemokine receptor 4 (CXCR4) on peripheral B cells. Blocking CXCR4 with AMD3100 markedly impaired BM migration, establishing the pathway’s functional necessity. This work proposed a model in which the BM acts as a transient immune reservoir during stress, conserving energy by sequestering B cells in a protected niche while prioritizing thermogenesis. This paradigm offers new insight into homeostatic immune regulation and potential therapeutic strategies for B-cell-mediated autoimmune disease.

## 1. Introduction

The continuous and coordinated trafficking of immune cells among the peripheral blood, lymphoid organs, and various tissues is fundamental to the maintenance of immunological homeostasis and the execution of effective responses to infection [1]. This dynamic redistribution, often referred to as recompartmentalization, ensures that specialized cell populations are positioned in appropriate physiological niches to receive essential survival signals or provide systemic surveillance against pathogens [1,2,3]. Current research has predominantly focused on the effects of pathological factors, such as inflammatory chemokines and cytokines that drive cell migration during acute infection or chronic disease. Our understanding of how homeostatic or ambient environmental stimuli regulate the steady-state distribution of immune populations remains substantially limited.

Ambient cold exposure constitutes a systemic, reversible, and nonpathological stimulus that profoundly influences physiological homeostasis. Cold-induced activation of the sympathetic nervous system modulates the bone marrow niche, thereby affecting populations such as hematopoietic stem cells [1]. In addition, cold stress elicits marked hemodynamic responses, including hypertension mediated through the renin–angiotensin–aldosterone system and elevated levels of circulating steroid hormones, particularly glucocorticoids [1,4]. Glucocorticoids regulate metabolism, immune activation, and leukocyte redistribution by directing the efflux or influx of cells between primary reservoirs, the spleen, and the bone marrow in a context-dependent manner [1,5,6,7,8,9,10]. For instance, circadian rhythms and stress-induced glucocorticoid secretion shape immune function [10,11,12,13,14]. However, the precise role of cold-induced glucocorticoids in directing leukocyte dynamics remains unknown. Although cold exposure has been linked to leukocytopenia, the specific leukocyte subsets most affected in peripheral blood under these conditions have yet to be conclusively identified.

At the molecular level, stress-induced B-cell homing in a young adult male mouse model was found to be closely governed by C-X-C chemokine receptor 4 (CXCR4) signaling. CXCR4 is a central regulator of immune cell trafficking, critical for the retention of B-lineage cells and other leukocytic precursors within the bone marrow microenvironment [9]. Although CXCR4 is well recognized for mobilizing neutrophils during emergency granulopoiesis [15], its role in the sequestration of mature B cells under environmental stress remains less defined. Pathological leukocyte retention in the bone marrow, termed myelokathexis, is a hallmark of warts, hypogammaglobulinemia, infections, and myelokathexis syndrome caused by CXCR4 gain-of-function mutations [16]. Beyond B cells, diverse hematopoietic and stromal populations—including T cells, neutrophils, monocytes, mesenchymal stem cells, malignant hematopoietic cells, and engineered CXCR4-expressing cells—depend on CXCR4 for bone marrow homing or infiltration in distinct contexts [2,3,9,17,18,19,20,21]. These observations underscore CXCR4 as an anchoring force that regulates immune cell distribution between circulation and bone marrow. Nevertheless, whether CXCR4 contributes to cold-induced leukocytopenia, B-cell reduction in peripheral blood, and subsequent bone marrow homing remains to be clarified.

In this study, we observed that the B cells in peripheral blood were reduced under cold exposure. This finding raises the possibility that systemic signaling pathways may be involved in coordinating the rapid reorganization of immune cell populations during cold stress. One possibility is that environmental cold stress could initiate the regulatory responses mediated by hormonal signals, such as increased glucocorticoids, which may contribute to the redistribution of B cells in circulation. The potential mechanisms underlying these processes are explored and discussed in the following sections.

## 2. Results

### 2.1. Ambient Cold Exposure and Dexamethasone Treatment Induce Systemic Leukocytopenia and Splenic Weight Reduction

**Figure 1:**
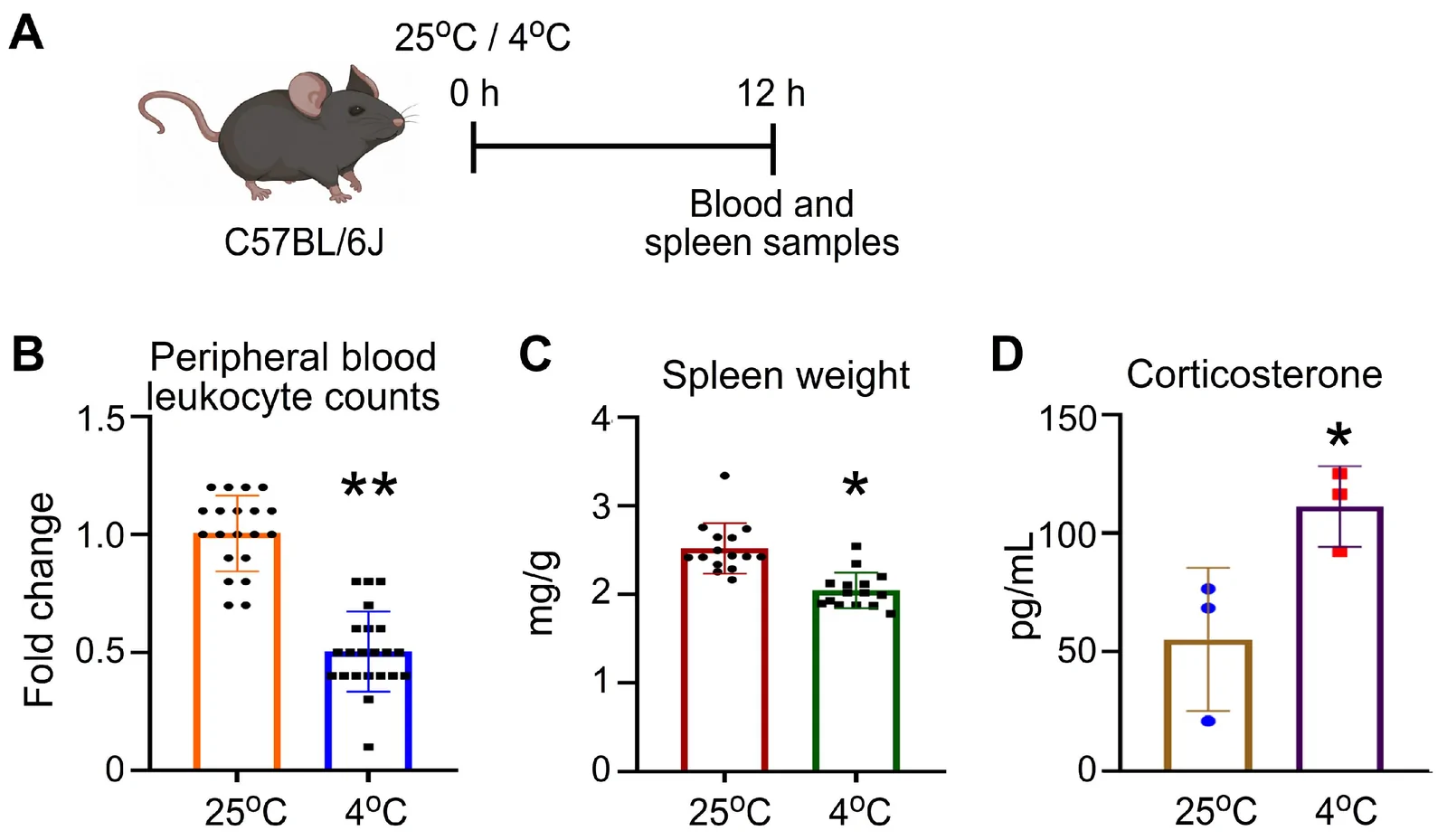
Cold exposure induced leukocytopenia and reduced spleen weight in mice. (**A**) Experimental design: C57BL/6J male mice (8–12 weeks old) were housed at two per cage and exposed for 12 h to either room temperature (25 °C) or cold (4 °C) prior to sample collection. (**B**) Peripheral blood leukocyte counts after 12 h of cold exposure compared with those of controls. Values for the 25 °C groups were normalized to onefold. (**C**) Spleen weight normalized to body weight in cold-exposed vs. control mice. (**D**) Circulating corticosterone levels in mice with or without cold exposure. Data are shown as mean ± SD from at least three independent experiments ((**B**): for 25 °C *n* = 21, and for 4 °C *n* = 22; (**C**): *n* = 15; (**D**): *n* = 3; each dot represents an individual mouse from pooled experiments). Statistical significance: * *p* < 0.05, ** *p* < 0.01.

To investigate how environmental and hormonal stressors affect the systemic distribution of immune cells, we first evaluated the effects of ambient cold stress. C57BL/6J male mice were exposed to a 4 °C environment for 12 h, while control groups were maintained at 25 °C (Figure 1A). Our results demonstrated that cold exposure markedly reduced the total leukocyte count in the peripheral blood compared with that in the room-temperature control (Figure 1B).

Peripheral immune cell depletion arises through three principal mechanisms: (1) cell death, (2) homing to peripheral lymphoid organs such as the spleen, or (3) homing to the bone marrow. Although the rapid clearance of B cells from the circulation strongly suggests active redistribution, alternative explanations are also available, including transient adhesion to the vascular endothelium or nonspecific transendothelial migration into peripheral tissues. To clarify the destination of these cells, we conducted adoptive-transfer and tissue-specific tracking assays.

Analysis of the spleen revealed that cold exposure reduced spleen weight relative to body weight (Figure 1C), rather than increasing it as expected given the accumulation of B cells in this organ. While splenic contraction alone cannot exclude sequestration, subsequent immunophenotyping (detailed below) demonstrated stable B-cell percentages within the contracting spleen, indicating that the absolute splenic B-cell pool diminished rather than expanded. Thus, the cleared circulating B cells did not accumulate in the spleen but instead reflected a systemic redistribution of lymphocytes. Consistent with stress responses, cold exposure also markedly elevated circulating corticosterone levels (Figure 1D), suggesting HPA axis activation.

**Figure 2:**
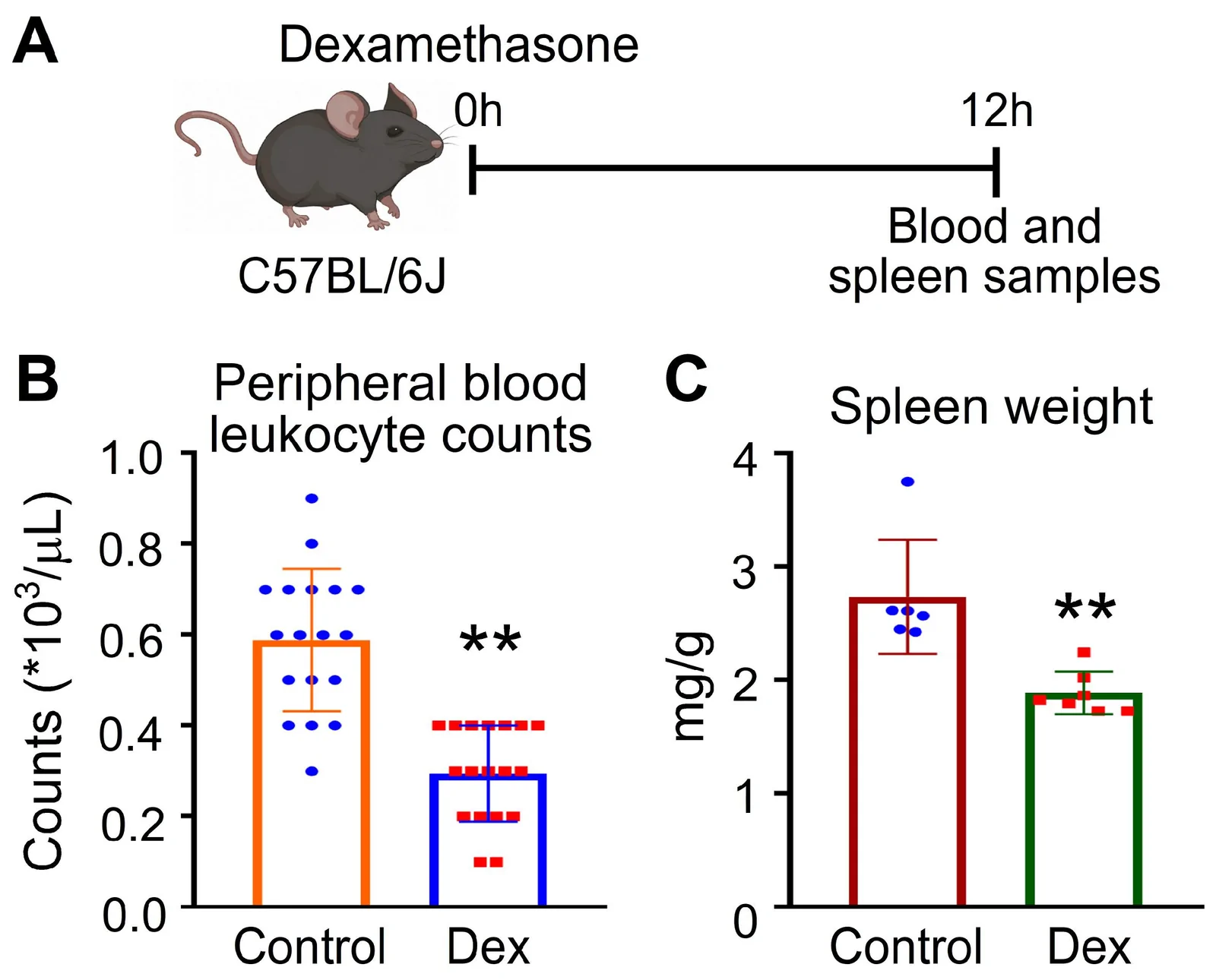
Dexamethasone treatments induced changes in leukocyte counts and spleen weight. (**A**) Experimental timeline: C57BL/6J male mice (8–12 weeks old) were injected intraperitoneally with 2 mg/kg dexamethasone (Dex) or saline (control) and maintained at 25 °C for 12 h before analysis. (**B**) Peripheral blood leukocyte counts measured 12 h post-injection. (**C**) Spleen weight normalized to body weight at 12 h post-injection. Data are presented as mean ± SD from three independent experiments ((**B**), *n* = 18; (**C**), *n* = 6). Each dot represents an individual mouse from pooled experiments. Statistical significance: ** *p* < 0.01.

Cold stress elicits strong hormonal responses. Mice were injected with dexamethasone (2 mg/kg) and evaluated after 12 h to determine whether these effects could be mimicked pharmacologically (Figure 2A). Similar to cold stress, dexamethasone treatment induced marked leukocytopenia in peripheral blood (Figure 2B) and markedly reduced spleen weight percentage (Figure 2C). These findings demonstrate that physical (cold) and hormonal (dexamethasone) stressors drive the systemic depletion of peripheral leukocytes and splenic mass, motivating further investigation into cell survival mechanisms and alternative homing sites such as the bone marrow.

### 2.2. Peripheral Leukocyte Depletion Is Not Driven by Cell Death

Cold exposure-induced leukocytopenia is highly reversible [22,23]. Evidence from experienced winter swimmers and cold-water immersion studies indicates that low water temperatures do not cause substantial leukocyte death [23,24,25]. To assess whether the leukocytopenia and reduced splenic mass observed in our mouse model can be attributed to leukocyte death, we performed a viability analysis using the zombie NIR kit, an amine-reactive fluorescent dye that identifies cells with damaged membranes. We first identified leukocyte and splenocyte populations based on forward and side scatter (FSC/SSC) profiles and subsequently analyzed them for fluorescent shifts indicative of cell death.

Consistent with previous reports [23,24,25], our analyses revealed that neither 12 h of cold exposure (Figure S1A–D) nor dexamethasone injection markedly induces cell death in either peripheral blood leukocytes or splenocytes (Figure S1E–H). Quantitative analysis showed that the ratio of live to dead cells remained stable across treatment and control groups, indicating that the total percentage of dead cells did not increase following these stimuli (cold exposure: Figure S1C,D; dexamethasone treatments: Figure S1G,H). These findings suggest that the peripheral depletion of immune cells is likely not caused by cell death. However, the evaluation of viability solely within the surviving circulating pool is insufficient to completely rule out stress-induced cell death. In physiological settings, cells undergoing rapid apoptosis under acute stress can be sequestered and cleared almost immediately by the reticuloendothelial system in organs such as the liver and spleen, thus preventing their detection as dead cells in peripheral blood. To definitively resolve whether the observed lymphopenia stems from systemic cell destruction or active recompartmentalization, we tracked the physical destination and recovery of B cells via quantitative adoptive-transfer assays.

### 2.3. Cold-Induced Stimuli Trigger a Reduction in Circulating B-Cell Counts

**Figure 3:**
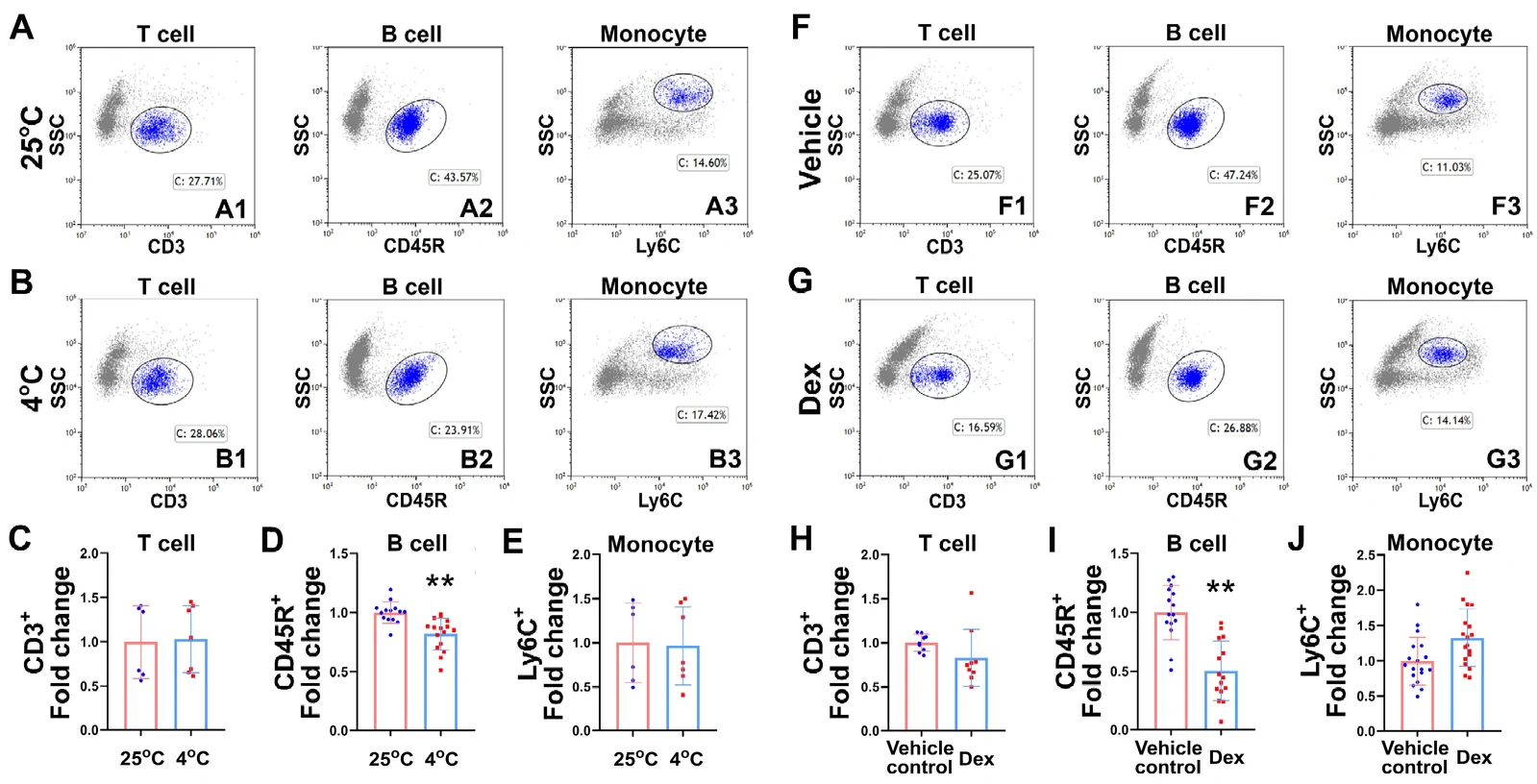
Peripheral B-cell counts were markedly downregulated after cold exposure and dexamethasone injections in mice. (**A**–**E**) Comparison of room temperature (25 °C; **A1**–**A3**) vs.cold exposure (4 °C; **B1**–**B3**). (**F**–**J**) Comparison of vehicle control (**F1**–**F3**) vs. dexamethasone (Dex; **G1**–**G3**) treatment. (**A**,**B**,**F**,**G**) Representative flow-cytometric gating of peripheral blood leukocytes. Elliptical gates delineate major subsets, with blue-colored populations indicating the gated cells used for analysis: Sub-panels (**A1**,**B1**,**F1**,**G1**): T cells (CD3^+^). Subpanels (**A2**,**B2**,**F2**,**G2**): B cells (CD45R^+^). Subpanels (**A3**,**B3**,**F3**,**G3**): monocytes (Ly6C^+^). Panels (**A**,**F**) show control conditions (25 °C and vehicle), and panels (**B**,**G**) show stress conditions (4 °C and Dex). (**C**,**H**) Quantification of T-cell percentages. (**D**,**I**) Quantification of B-cell percentages, showing significant downregulation under stress. (**E**,**J**) Quantification of monocyte percentages. Values for the 25 °C and vehicle groups were normalized to onefold (**C**–**E**,**H**–**J**). Data are presented as mean ± SD from at least three independent experiments ((**C**): for 25 °C *n* = 6, and for 4 °C *n* = 7; (**D**): for 25 °C *n* = 14, and for 4 °C *n* = 16; (**E**): for 25 °C *n* = 6, and for 4 °C *n* = 7; (**H**): *n* = 9; (**I**): *n* = 15; (**J**): *n* = 18). Each dot represents one mouse from pooled experiments. Statistical significance: ** *p* < 0.01.

Considering that cell death and splenic sequestration were excluded, we next investigated whether peripheral immune cells actively migrate to the bone marrow. Mice were subjected either to cold exposure (4 °C vs. 25 °C; Figure 3A–E) or dexamethasone treatment (vehicle vs. Dex; Figure 3F–J). After 12 h, we quantified the relative proportions of circulating T cells, B cells, and monocytes (Figure 3C–E,H–J). Among these leukocyte subsets, only B cells showed a pronounced reduction following cold exposure or dexamethasone administration (Figure 3D,I).

### 2.4. Cold Exposure and Dexamethasone Drive the Sequestration of Circulating B Cells in the Bone Marrow

**Figure 4:**
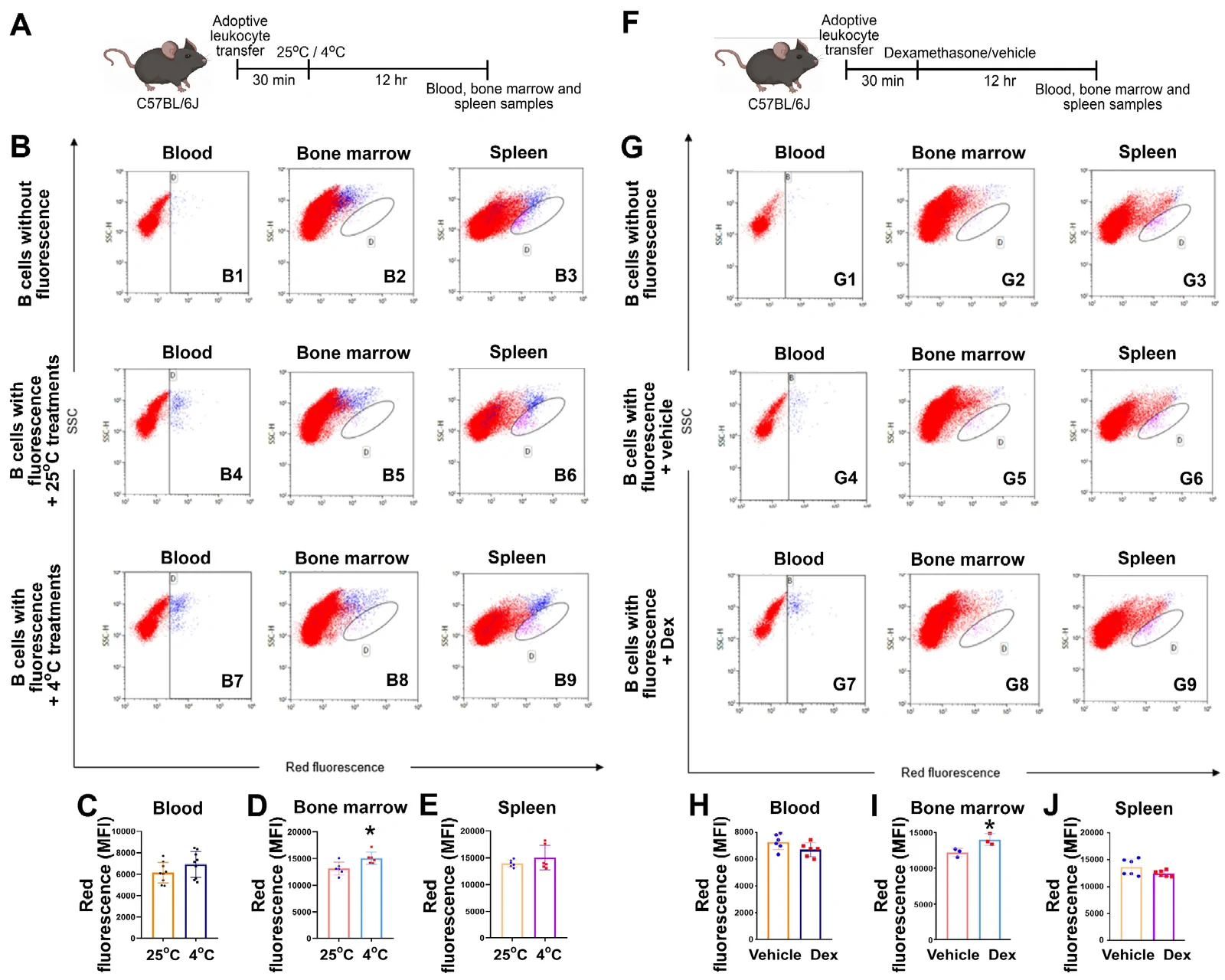
Transfer of red fluorescence-expressing B cells homing to bone marrow after cold exposure and dexamethasone injections in mice. (**A**,**F**) Experimental timeline: Red-fluorescent (tdTomato^+^) leukocytes were adoptively transferred into C57BL/6J recipient mice. After 30 min of circulation, the mice were either exposed to cold (4 °C vs. 25 °C) or injected with dexamethasone (Dex, 2 mg/kg vs. vehicle saline). Peripheral blood, bone marrow, and spleens were collected 12 h later. (**B**,**G**) Flow-cytometric gating strategy: Panels (**B1**–**B3**,**G1**–**G3**) show wild-type B cells (nonfluorescent) as background controls alongside tomato-red transgene-expressing B cells. Samples were analyzed from peripheral blood (**B1**,**B4**,**B7**,**G1**,**G4**,**G7**), bone marrow (**B2**,**B5**,**B8**,**G2**,**G5**,**G8**), and spleens (**B3**,**B6**,**B9**,**G3**,**G6**,**G9**) across treatment groups (25 °C, 4 °C, vehicle, Dex). Elliptical gates (Gate D) was established to isolate red-fluorescent donor populations above baseline background events (e.g., cell size variation, autofluorescence). In flow-cytometric dot plots, red-colored events represent cell populations with low background fluorescence, whereas blue-colored events denote cell populations displaying higher red fluorescence intensity; the additional population appearing at Gate D in treated groups (**B4**–**B9**,**G4**–**G9**) represents true homed donor cells and was used for quantification. (**C**,**H**) Quantified relative levels of red-fluorescent B cells in peripheral blood. (**D**,**I**) Quantified relative levels of red-fluorescent B cells in bone marrow. (**E**,**J**) Quantified relative levels of red-fluorescent B cells in spleens. A minimum of 50,000 events (blood) or 100,000 events (marrow/spleen) were acquired per replicate. Data are presented as individual biological replicates (dots), each representing one mouse from pooled independent experiments, with mean ± SD shown. Sample sizes: (**C**), *n* = 9; (**D**,**E**,**H**,**J**), *n* = 6; (**I**), *n* = 3. Statistical significance: * *p* < 0.05.

Building on the findings that stress-induced leukocytopenia cannot be attributed to cell death and peripheral blood B cells are one of the major cell types to be affected by cold exposure, we performed the adoptive transfer of red-fluorescent leukocytes [total leukocyte fractions including B cells from B6.129-(Cg)-Gt(ROSA)26Sor^tm4(ACTB-tdTomato,-EGFP)Luo/JNarl mice] to validate the homing patterns of peripheral blood B cells across stress models. Labeled leukocytes were intravenously introduced into recipient mice and allowed to circulate for 30 min before cold exposure (4 °C) or dexamethasone injection. After 12 h, the mean fluorescence intensity (MFI) of the red signal and B-cell markers were quantified to track the destination of transferred cells (4 °C vs. 25 °C, Figure 4A–E; vehicle vs. Dex, Figure 4F–J). To precisely identify the newly homed donor cells and eliminate nonspecific background signals, we compared the gating profiles of the bone marrow across control and treatment groups (Figure 4(B2,B5,B8)). In the nonfluorescent control group (Figure 4(B2)), the red color represents cell populations with low red fluorescence, and the blue color represents cell populations with relatively high red fluorescence. A small subset of nonfluorescent cells naturally fell into this high-red-fluorescence region because of physical properties (such as large cell size and autofluorescence), forming a nonspecific background in the left-upper region. This background distribution was virtually identical across the graphs in Figure 4(B2,B5,B8). Accordingly, Gate D was established to set a threshold immediately above this baseline background. This background-exclusion strategy has allowed us to visualize and quantify the newly appeared, true red-fluorescent donor B cells (the “extra population”) that homed to the bone marrow niche under cold exposure (Figure 4(B8), Gate D) compared with that at room temperature (Figure 4(B5), Gate D) and the nonfluorescent control baseline (Figure 4(B2), Gate D). The marked increase in red-fluorescent MFI within the bone marrow (Figure 4D,J) provides strong evidence that physical and hormonal stressors drive the redistribution of circulating B cells into the bone marrow niche.

### 2.5. B Cells Are One of the Principal Leukocyte Populations Homing to the Bone Marrow Under Stress

**Figure 5:**
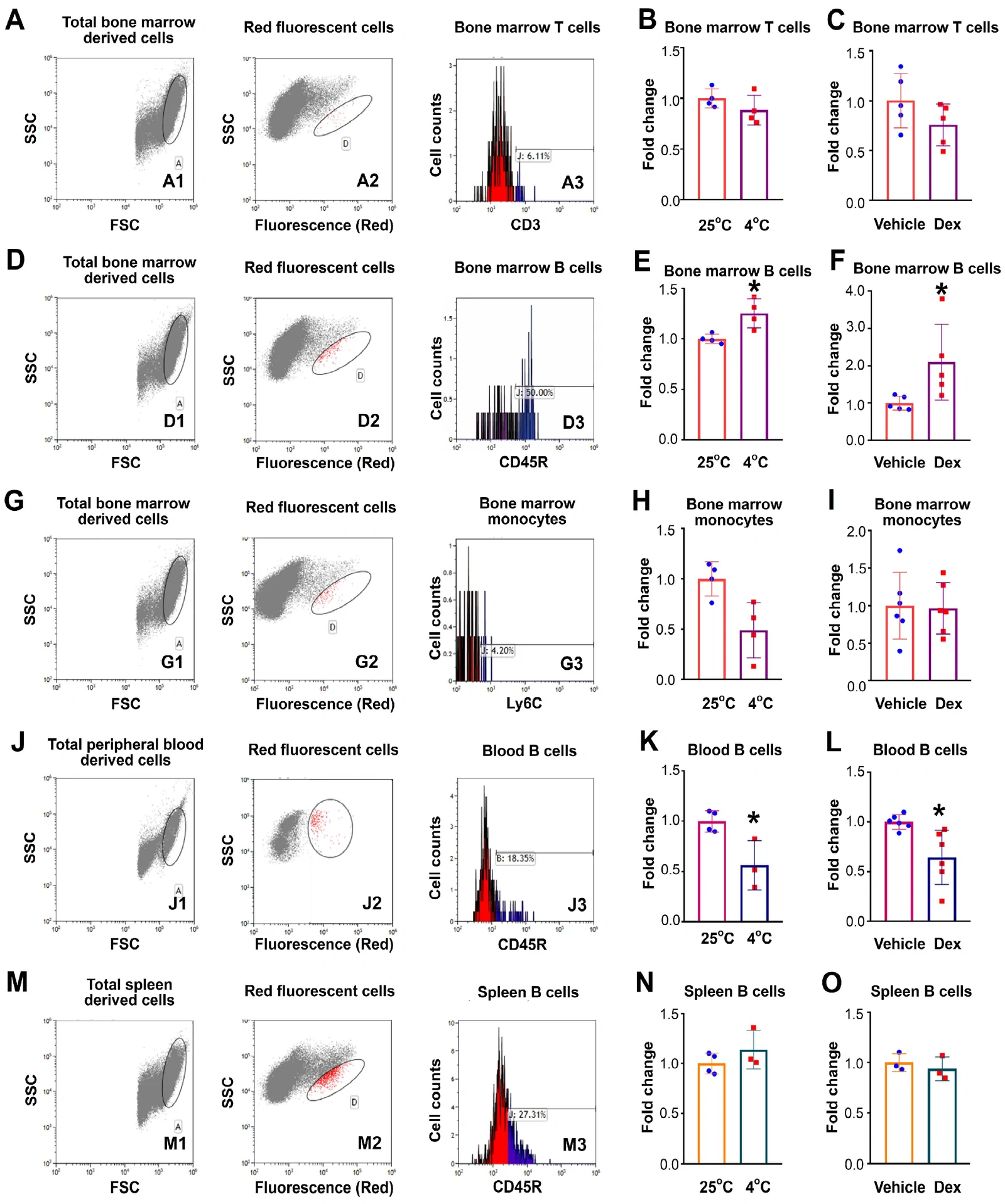
Circulating B cells are one of the major leukocyte populations homing to bone marrow after cold exposure and dexamethasone injections in mice. (**A**,**D**,**G**,**J**,**M**) Representative flow-cytometric gating strategy, organized in parallel lineage rows for clear signal separation: Panels (**A1**,**D1**,**G1**,**J1**,**M1**): leukocyte identification using forward scatter (FSC) vs. side scatter (SSC). Panels (**A2**,**D2**,**G2**,**J2**,**M2**): gating by SSC and red fluorescence to isolate adoptively transferred cells. Panels (**A3**,**D3**,**G3**,**J3**,**M3**): lineage staining for T cells (CD3; **A1**–**A3**), B cells (CD45R; **D1**–**D3**, **J1**–**J3**, **M1**–**M3**), and monocytes (Ly6C; **G1**–**G3**). In these histograms, red regions denote cells with absent or low lineage marker expression, whereas purple regions denote cells with high lineage marker expression, relative to isotype control antibody background. (**B**,**C**,**E**,**F**,**H**,**I**) Quantification of red-fluorescent T cells, B cells, and monocytes sequestered in the bone marrow. (**K**,**L**,**N**,**O**) Quantification of red-fluorescent B cells in peripheral blood and spleens. (**B**–**O**) Quantification of relative homing fold-changes for transferred tdTomato^+^ donor T cells, B cells, and monocytes in recipient tissue compartments, expressed as the fold-change in lineage percentages within the gated donor (tdTomato^+^) population relative to controls. Values for the 25 °C and vehicle groups were normalized to onefold (**B**,**C**,**E**,**F**,**H**,**I**,**K**,**L**,**N**,**O**). Data are presented as individual biological replicates (dots), each representing one mouse from pooled independent experiments, with mean ± SD shown. Sample sizes: (**B**,**E**,**H**): *n* = 4; (**C**,**F**,**I**): *n* = 5; (**K**,**N**): for 25 °C *n* = 4, and for 4 °C *n* = 3; (**L**): *n* = 6; O: *n* = 3. Statistical significance: * *p* < 0.05.

To characterize the specific leukocyte subsets responsible for the observed redistribution, we analyzed the composition of the red-fluorescent cell population (tdTomato^+^) sequestered in the bone marrow. To ensure quantitative clarity, homing efficiency was evaluated by determining the relative percentage of lineage-specific markers (CD3 for T cells, CD45R for B cells, and Ly6C for monocytes) strictly within the gated donor (tdTomato^+^) compartment, expressed as fold-change relative to controls (Figure 5A–O).

Our analysis revealed that circulating B cells represent one of the major leukocyte populations that home back to the bone marrow niche following these stimuli. In this mixed-population transfer model (~5 × 10^6^ total leukocytes), the relative fold-change in donor B cells within the bone marrow donor pool showed a pronounced increase (Figure 5E,F), whereas donor T cells and monocytes displayed no significant enrichment (Figure 5B,C,H,I). This selective enrichment of B cells within the transferred compartment—coupled with their corresponding reduction in peripheral blood (Figure 5K,L)—demonstrates a cell-type-specific homing preference toward the bone marrow microenvironment under physiological stress.

In the spleens of recipient mice subjected to cold stress, the percentage of transferred tdTomato^+^ CD45R^+^ B cells remained unchanged compared with that in room-temperature controls (Figure 5N). Given the substantial decrease in total spleen weight (Figure 1C)—which is directly proportional to total splenic cellularity—the combination of a reduced splenic mass and a constant donor B-cell percentage indicates a net decrease in the absolute number of homed donor B cells within the spleen. This quantitative deduction supports our conclusion that the stress-induced B-cell clearance from the blood is not due to passive splenic trapping but is preferentially directed toward the bone marrow.

### 2.6. Cold Exposure and Dexamethasone Induce CXCR4 Expression Specifically on B Cells

**Figure 6:**
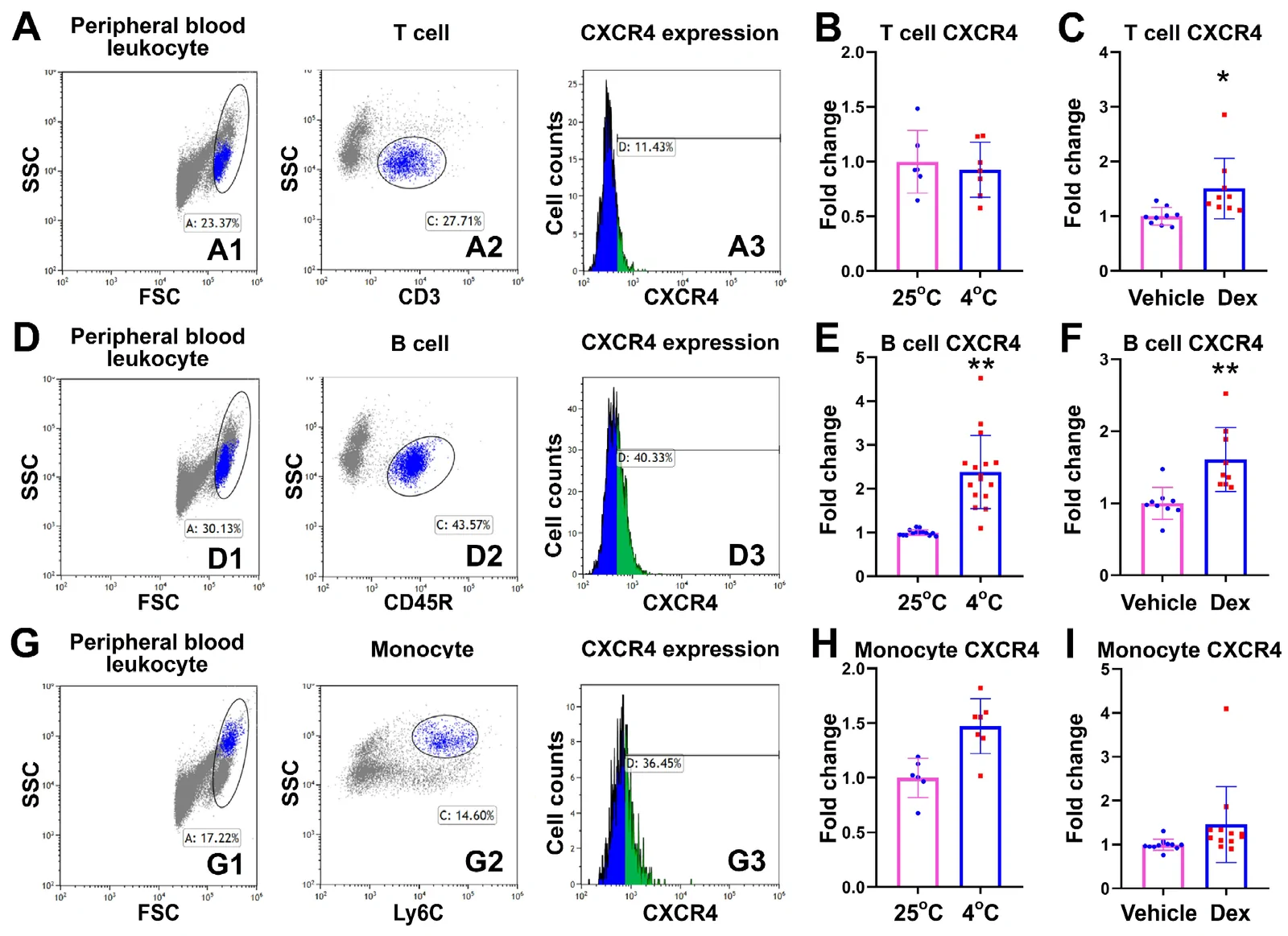
Cold exposure and dexamethasone treatments upregulate CXCR4 on B cells. Peripheral blood CXCR4 expression was analyzed in (**A**–**C**) T cells, (**D**–**F**) B cells, and (**G**–**I**) monocytes. Flow-cytometric gating showing separate analysis pathways for each leukocyte lineage (**A**,**D**,**G**). Panels (**A1**,**D1**,**G1**): identification of leukocytes from purified cells using forward scatter (FSC) and side scatter (SSC). Panels (**A2**,**D2**,**G2**): subdivision into T cells, B cells, and monocytes using lineage markers (CD3, CD45R, Ly6C). Panels (**A3**,**D3**,**G3**): evaluation of CXCR4 surface expression (histograms) within each subset. In these histograms, blue regions denote low-CXCR4-expressing cells, whereas green regions denote high-CXCR4-expressing cells, relative to isotype control antibody background. T cell analyses (**A1**–**A3**), B cell analyses (**D1**–**D3**), monocyte analyses (**G1**–**G3**). Quantitative analyses: (**B**,**C**) CXCR4 expression on T cells. (**E**,**F**) CXCR4 expression on B cells, showing pronounced stress-induced upregulation. (**H**,**I**) CXCR4 expression on monocytes. Comparisons: (**B**,**E**,**H**) 25 °C vs. 4 °C groups; (**C**,**F**,**I**) vehicle vs. dexamethasone (Dex) groups. Values for the 25 °C and vehicle groups were normalized to onefold (**B**,**C**,**E**,**F**,**H**,**I**). Data are presented as individual biological replicates (dots), each representing one mouse from pooled independent experiments, with mean ± SD shown. Sample sizes: (**B**,**H**): for 25 °C *n* = 6, and for 4 °C *n* = 7; (**E**): for 25 °C *n* = 14, and for 4 °C *n* = 16; (**C**,**F**): *n* = 9; (**I**): *n* = 12. Statistical significance: * *p* < 0.05, ** *p* < 0.01.

To elucidate the molecular mechanism driving B-cell redistribution, we analyzed the expression of CXCR4, a key chemokine receptor known for its role in bone marrow homing. We identified peripheral blood T-cell, B-cell, and monocyte populations using lineage-specific markers (CD3, CD45R, and Ly6C) and quantified their surface CXCR4 expression following stress stimuli (4 °C vs. 25 °C, Figure 6B,E,H; vehicle vs. Dex, Figure 6C,F,I).

Our analysis revealed that cold exposure and dexamethasone treatments markedly upregulated CXCR4 on B-cell surfaces (Figure 6E,F). By contrast, the percentage of CXCR4 expression on T cells and monocytes did not show the same level of consistent, robust induction (Figure 6B,C,H,I). These findings suggest that the B-cell-specific upregulation of CXCR4 provides the necessary molecular drive for these cells to exit the circulation and home back to the bone marrow niche under stress conditions.

### 2.7. Blocking CXCR4 Prevents B-Cell Homing to the Bone Marrow During Cold Exposure and Dexamethasone Treatment

To verify the functional role of CXCR4 in B-cell redistribution, we conducted a blockade experiment using AMD3100, a selective CXCR4 antagonist. Red-fluorescent leukocytes were adoptively transferred into recipient mice, followed by the intraperitoneal injection of either AMD3100 or saline immediately prior to exposure to 4 °C/25 °C environments or dexamethasone administration.

**Figure 7:**
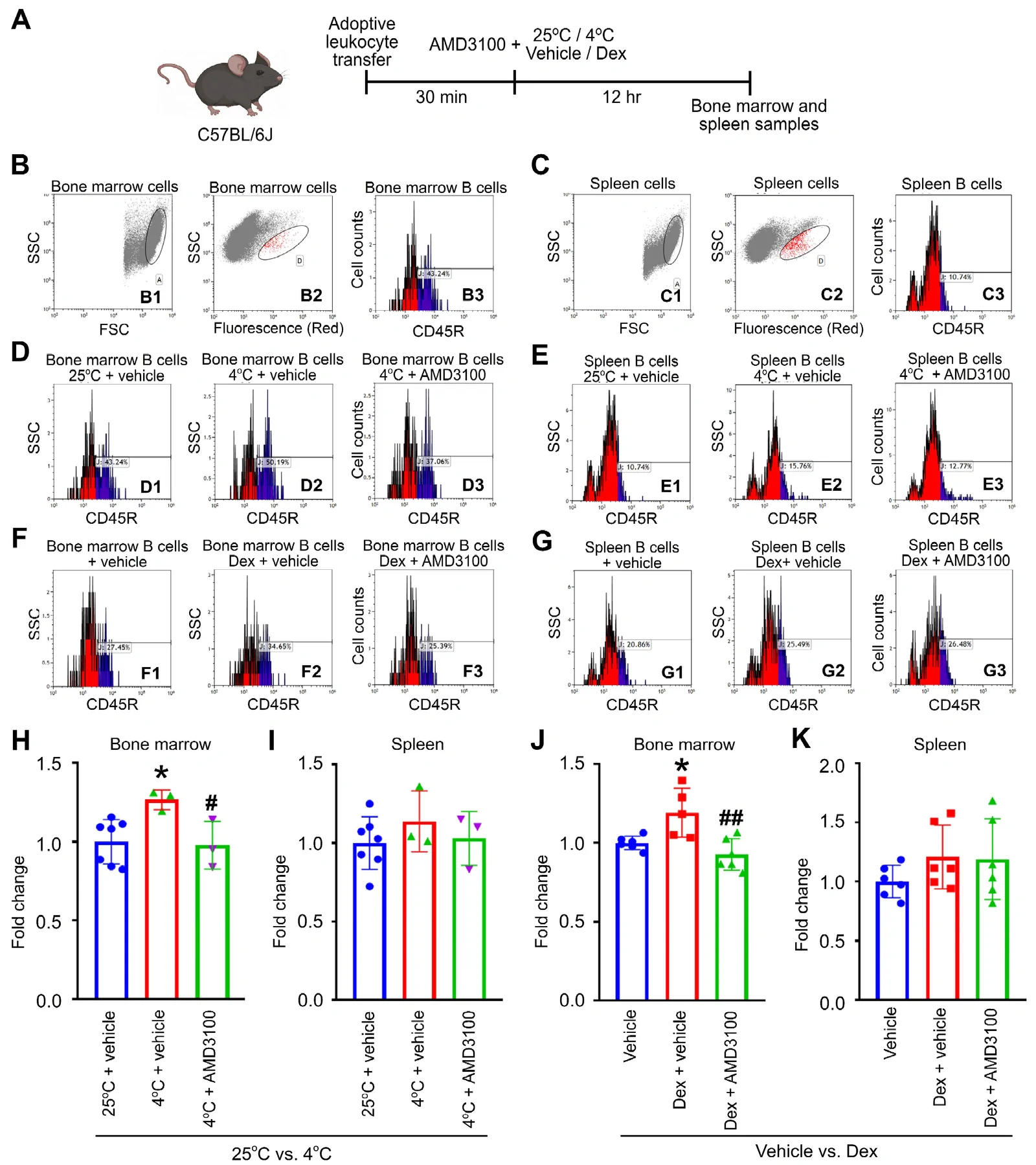
AMD3100 prevents B-cell re-entry into bone marrow during cold exposure and dexamethasone treatment. (**A**) Schematic of the CXCR4 blockade experiment: Red-fluorescent leukocytes were adoptively transferred into C57BL/6J male mice and allowed to circulate for 30 min. The mice then received intraperitoneal injections of either AMD3100 or saline immediately before 12 h exposure to 4 °C/25 °C environments or treatment with vehicle/dexamethasone (Dex). (**B**–**G**) Representative flow-cytometric gating strategies and profiles: Panels (**B1**,**C1**) identify the leukocyte population (Gate A) using forward scatter (FSC) and side scatter (SSC) in recipient bone marrow (**B1**–**B3**) and spleen (**C1**–**C3**). Panels (**B2**,**C2**) define the adoptively transferred donor leukocytes (Gate D) based on SSC vs. red fluorescence. Panels (**B3**,**C3**) show CD45R^+^ B cells (Gate J) within the gated donor population. Panels (**D1**–**D3**), and (**E1**–**E3**) (cold stress) and (**F1**–**F3**), and (**G1**–**G3**) (dexamethasone treatment) depict representative plots of CD45R^+^ B-cell levels (Gate J) in the bone marrow (**D**,**F**) and spleen (**E**,**G**) across treatment groups (25 °C + vehicle, 4 °C + vehicle, 4 °C + AMD3100, Dex + vehicle, Dex + AMD3100). (**D**–**G**) In the histograms, red regions denote cells with absent or low CD45R expression (non-B cells), whereas purple regions denote cells with high CD45R expression (B cells). (**H**–**K**) Quantified relative fold-changes in homed donor CD45R^+^ B cells (gated on tdTomato^+^ donor population) in recipient bone marrow (**H**,**J**) and spleens (**I**,**K**) across treatment groups. Values for the 25 °C + vehicle and vehicle groups were normalized to onefold (**H**–**K**). Data are presented as individual biological replicates (dots), each representing one mouse from pooled independent experiments, with mean ± SD shown Sample size: (**H**,**I**): for 25 °C + vehicle *n* = 7, for 4 °C + vehicle *n* = 3, and for 4 °C + AMD3100 *n* = 3; (**J**,**K**): *n* = 6, except J Dex, for which *n* = 5. Statistical comparisons: * *p* < 0.05 for 4 °C vs. 25 °C and vehicle vs. Dex; # *p* < 0.05 for 4 °C vs. 4 °C + AMD3100; ## *p* < 0.01 for Dex + vehicle vs. Dex + AMD3100.

These results demonstrated that AMD3100 markedly inhibited the return of B cells to the bone marrow under cold exposure and dexamethasone treatment (Figure 7). Representative flow-cytometric analysis showed that adoptively transferred tdTomato^+^ donor cells (gated on FSC vs. red fluorescence, Figure 7B,C) migrated to the recipient bone marrow and spleen. In the bone marrow, a prominent peak of CD45R^+^ B cells was observed under cold stress (Figure 7(D2)) or dexamethasone challenge (Figure 7(F2)), which was robustly suppressed upon the pharmacological blockade of CXCR4 by AMD3100 (Figure 7(D3,F3)). Quantitative analysis revealed a pronounced reduction in red-fluorescent B cells homing to the bone marrow, but not the spleen, in AMD3100-treated mice compared with those in saline-treated controls exposed to cold (Figure 7H,I). Similarly, B-cell homing in the bone marrow—but not the spleen—was markedly reduced in the AMD3100-treated mice compared with that in vehicle-treated controls under dexamethasone treatment (Figure 7J,K). These observations are consistent with the hypothesis that CXCR4 signaling plays an important role in stress-associated B-cell sequestration within the bone marrow.

### 2.8. Glucocorticoid Receptor Blockade Prevents Stress-Induced CXCR4 Upregulation on Circulating B Cells

To confirm that an elevated corticosterone level is directly responsible for driving the upregulation of surface CXCR4 on B cells, we performed an in vivo receptor blockade experiment using the competitive glucocorticoid and progesterone receptor antagonist mifepristone (RU486). Mice were intraperitoneally (i.p.) administered RU486 (mifepristone; 20 mg/kg), prepared in corn oil after dissolving in DMSO for 60 min (1 h) prior to placement in the cold chamber (4 °C).

**Figure 8:**
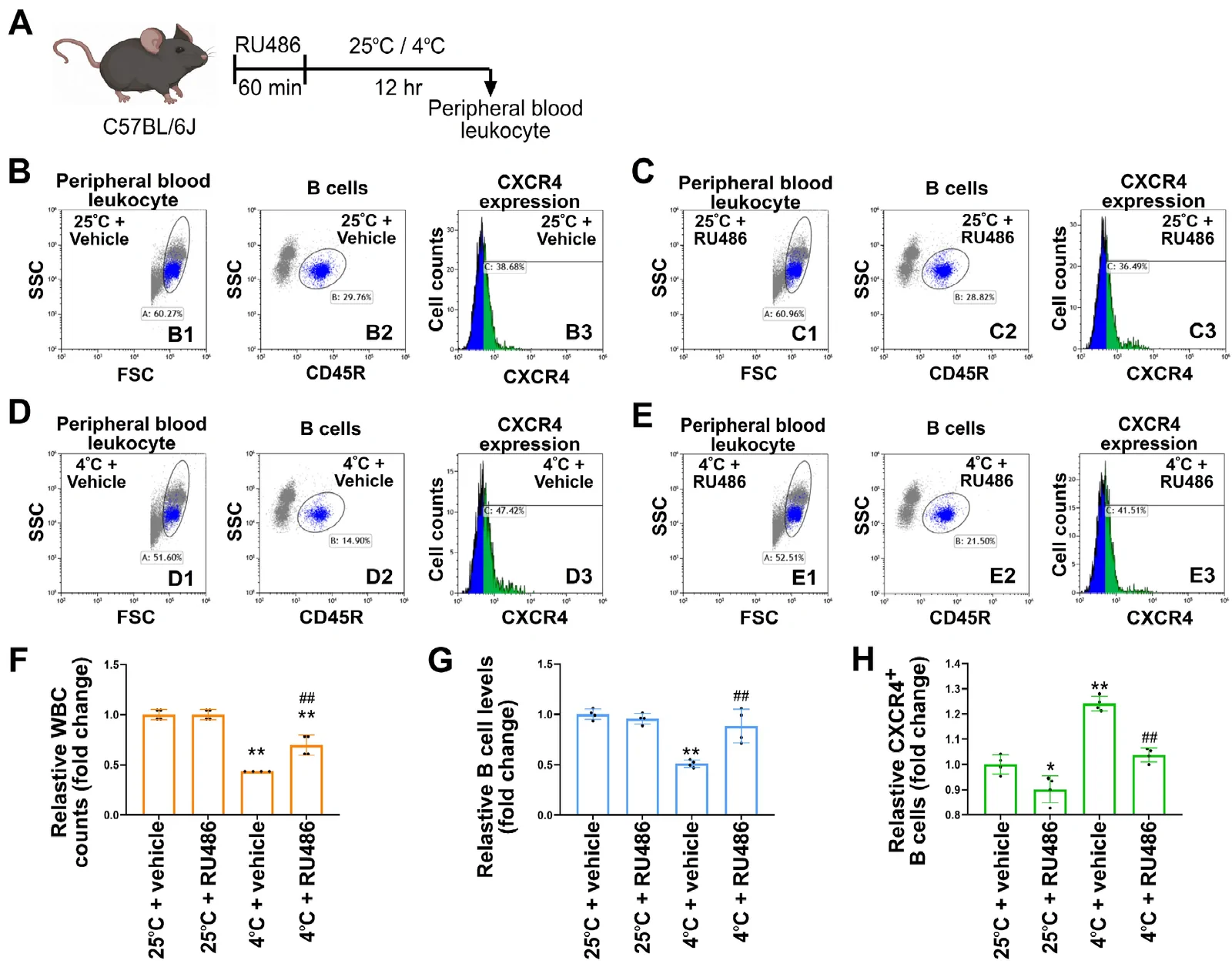
Glucocorticoid receptor blockade with RU486 prevents stress-induced CXCR4 upregulation on B cells. Male C57BL/6J mice were pretreated with vehicle or RU486 (20 mg/kg, i.p.) 60 min prior to 12 h exposure to room temperature (25 °C) or cold stress (4 °C). (**A**) Experimental design. (**B**–**E**) Representative flow-cytometric gating strategy for 25 °C + vehicle (**B**,**B1**–**B3**), 25 °C + RU486 (**C**,**C1**–**C3**), 4 °C + vehicle (**D**,**D1**–**D3**), and 4 °C + RU486 (**E**,**E1**–**E3**). Panels (**B1**,**C1**,**D1**,**E1**) identify leukocytes by forward scatter (FSC) and side scatter (SSC); (**B2**,**C2**,**D2**,**E2**) further define B cells by CD45R staining; (**B3**,**C3**,**D3**,**E3**) depict relative CXCR4 expression on B cells determined by antibody staining. In these histograms, blue regions denote low-CXCR4-expressing cells, whereas green regions denote high-CXCR4-expressing cells, relative to isotype control antibody background. (**F**–**H**) Quantitative analysis of peripheral blood leukocyte counts (**F**), B-cell frequencies (**G**), and B-cell surface CXCR4 expression (**H**). Values for the 25 °C + vehicle group were normalized to onefold (**F**–**H**). For pooled independent experiments, data are presented as individual replicates (dots), with mean ± SD (*n* = 4 per group, each dot representing one mouse). * *p* < 0.05, ** *p* < 0.01 vs. respective 25 °C groups; ## *p* < 0.01 vs. 4 °C + RU486 groups.

Flow-cytometric analysis of circulating PBMCs showed that cold stress markedly increased the CXCR4 expression (MFI) of CD45R+ B cells in the vehicle-pretreated mice. By contrast, pretreatment with the glucocorticoid receptor blocker RU486 completely abolished this upregulation, maintaining CXCR4 expression at levels comparable with those in the room-temperature control group (Figure 8). These findings demonstrate that stress-induced CXCR4 upregulation in peripheral B cells is actively mediated through a glucocorticoid receptor-dependent pathway.

### 2.9. Summary of the Regulatory Pathway for Stress-Induced B-Cell Homing

**Figure 9:**
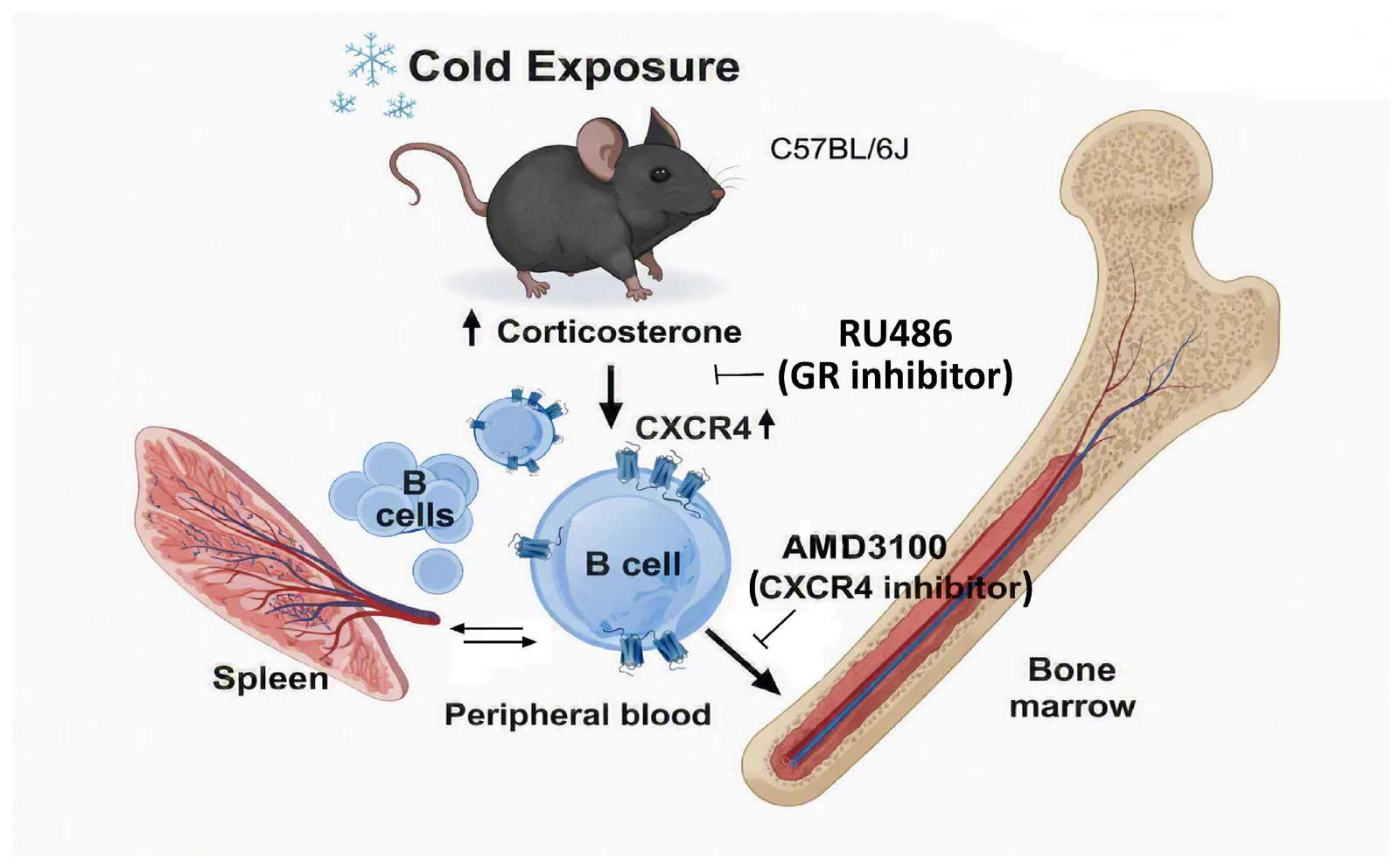
Proposed regulatory pathway for stress-induced B-cell homing. Schematic representation of the humoral and receptor-mediated mechanisms governing selective B-cell recompartmentalization under physiological stress. Exposure to ambient cold elevates circulating corticosterone, which acts through the glucocorticoid receptor (GR) to upregulate surface CXCR4 expression on peripheral CD45R^+^ B cells. Treatment with the GR antagonist RU486 suppresses this cold-induced CXCR4 elevation, supporting GR involvement. Enhanced CXCR4 expression facilitates directed migration of B cells from the circulation into the bone-marrow microenvironment, serving as a transient immune reservoir for metabolic resource conservation. This homing cascade can be pharmacologically interrupted at the receptor level by the CXCR4 antagonist AMD3100.

Our collective findings support a novel regulatory model for immune cell redistribution under environmental stress. As illustrated in Figure 9, cold exposure acts as a systemic trigger that leads to elevated corticosterone levels. This elevation in the hormonal signal (corticosterone) in peripheral B cells induces a marked increase in the expression of the homing receptor CXCR4. This high CXCR4 expression facilitates the migration of B cells from the peripheral blood back into the bone marrow, which serves as a transient immune reservoir. The inhibition of this process by AMD3100 further suggests that B-cell trafficking during cold stress is a regulated, receptor-mediated physiological adaptation designed to protect immune cell populations in a sequestered niche.

## 3. Discussion

This study demonstrates that ambient cold stress and dexamethasone treatment trigger rapid systemic leukocytopenia, characterized specifically by a reduction in circulating B cells and splenic mass. Utilizing cell viability assays and adoptive-transfer experiments with fluorescently labeled leukocytes, we established that this peripheral depletion is likely not a consequence of cell death but rather a directed redistribution of B lymphocytes to the bone marrow. Although our blood-based viability assays showed no increase in B-cell death, we must caution that the rapid phagocytic clearance of apoptotic cells in peripheral tissues could mask an underlying cytotoxic phenotype. Nevertheless, our adoptive-transfer and tracking data (Figure 4, Figure 5 and Figure 7) provide a strong counterargument to widespread apoptosis. By transferring a defined cohort of viable, fluorescently labeled donor cells, we demonstrated that the reduction in circulating donor B cells was quantitatively accompanied by selective, highly enriched accumulation of these identical donor B cells within the bone marrow niche. This bone marrow accumulation was completely reversed by the CXCR4 antagonist AMD3100, suggesting that the B-cell deficit in circulation is primarily driven by active, receptor-mediated homing to the bone marrow protective reservoir rather than systemic cell destruction. This phenomenon expands the “barracks to boulevards to battlefields” model of stress-induced immune cell trafficking [5]. Different from standard myeloid cell mobilization observed in many acute stressors [26,27,28], mature B cells respond to corticosterone elevation by homing to and are transiently sequestered within the bone marrow niche.

Splenic sequestration is a common pathway for transient leukocyte clearance [29,30]. Although we did not directly measure absolute splenocyte counts using a hemocytometer, the significant reduction in spleen weight, coupled with the stable percentages of transferred B cells (Figure 4 and Figure 5), mathematically argues against splenic accumulation. Given that absolute splenic B-cell numbers decrease when the total tissue mass shrinks and the B-cell fraction remains constant, the spleen does not serve as a reservoir for the cleared peripheral B cells. Nonetheless, we phrase this conclusion cautiously, acknowledging that subtle, localized endothelial adhesion within the splenic red pulp cannot be completely excluded without further high-resolution tissue imaging.

The molecular drive for this recompartmentalization was found to be governed by CXCR4 upregulation on the surface of B cells in a young adult male mouse model. While CXCR4 serves as a master regulator of bone marrow retention for various hematopoietic lineages, our findings highlight a divergent regulatory balance between B cells and myeloid cells. In emergency granulopoiesis, trafficking is often governed by a tug-of-war between CXCR4-mediated retention and CXCR2-mediated mobilization [31,32]. However, in our experimental model, environmental stress shifts this balance toward B-cell sequestration. The functional necessity of this receptor-mediated pathway was supported by the administration of the CXCR4 antagonist AMD3100, which effectively blocked stress-induced B-cell homing to the bone marrow. To resolve the key upstream regulatory mechanism, we introduced the glucocorticoid receptor antagonist RU486. Our results show that pre-emptive glucocorticoid receptor blockade effectively prevented cold-induced CXC4 upregulation in peripheral B cells. The suppression of CXCR4 upregulation, the primary molecular step for enabling bone marrow homing, supports the model where elevated glucocorticoids instruct B-cell redistribution through the GR–CXCR4 axis.

The glucocorticoid-driven redistribution of circulating lymphocytes to the bone marrow is a well-established paradigm [33,34], and CXCR4/CXCL12 signaling is recognized as a central mechanism for retaining hematopoietic precursors within marrow niches [35,36,37]. However, rapid and selective recompartmentalization of mature peripheral B cells—distinct from T cells and monocytes—in response to a naturalistic stressor such as ambient cold has not previously been described. We demonstrate that this B-cell-specific homing is mediated by glucocorticoid-dependent upregulation of surface CXCR4.

Pharmacological administration of dexamethasone at room temperature reproduced the cold-induced phenotype, driving CXCR4 expression and B-cell migration to the marrow (Figure 4, Figure 5 and Figure 7). Since ambient cold robustly activates the HPA axis and corticosterone release, it is plausible that other stressors, such as restraint, social conflict, or tissue trauma, that elevate glucocorticoids engage the same pathway. Thus, B-cell recompartmentalization appears less a cold-specific phenomenon than a fundamental neuroendocrine-immune reflex. Under diverse acute challenges, transient withdrawal of B cells into the marrow niche may shield adaptive lymphocytes from systemic stress and conserve metabolic energy for immediate survival needs.

From an evolutionary perspective, this transient sequestration represents a potential adaptive mechanism. Environmental stressors, particularly cold, impose high energetic demands that induce prioritization of thermogenesis. By relocating energy-intensive immune populations into a protected reservoir [23], the host conserves resources without permanently compromising adaptive immunity. This paradigm parallels leukocyte responses observed in fasting [38,39,40,41,42] and exercise [43,44,45,46,47,48,49]. Clinically, harnessing this mechanism may offer novel strategies for B-cell-mediated autoimmune diseases such as systemic lupus erythematosus and rheumatoid arthritis, by redirecting pathogenic cells from inflamed sites back to the marrow. Conversely, maladaptive sequestration—such as the accumulation of specialized B-cell subsets linked to age-related osteoporosis [50]—underscores the need for careful modulation of this axis.

This adaptive blueprint finds strong parallels in comparative immunology and the physiology of ectothermic vertebrates, such as fish, amphibians, and reptiles. In these species, seasonal cold exposure, artificial cold acclimation, and winter hibernation trigger “cold-induced lymphopenia,” characterized by rapid clearance of lymphocytes from the peripheral circulation [51,52,53]. This clearance does not stem from systemic cell destruction, but rather from the active, transient redistribution of circulating lymphocytes into protective tissues, such as the primary lymphoid organ the head kidney and the secondary organ the spleen in teleost fish, or the bone marrow and liver in amphibians and reptiles [54,55,56,57,58]. This temporary reallocation is hypothesized to shield vulnerable immune cells from metabolic exhaustion and environmental stressors, conserving energy when systemic metabolic rates are severely depressed. By translating this concept to our mammalian model, we reveal that the glucocorticoid-driven, CXCR4-mediated homing of B cells to the bone marrow under cold exposure suggests that endotherms have retained and adapted this ancient vertebrate survival mechanism to temporarily prioritize high-energy thermogenesis over peripheral immune surveillance without permanently depleting their immunological arsenal.

### Limitations and Future Directions

Our finding that AMD3100 treatment blocks B-cell accumulation in the bone marrow supports the involvement of the CXCR4 pathway; however, several caveats must be acknowledged. AMD3100 broadly alters leukocyte trafficking, mobilizing neutrophils and hematopoietic progenitors from the bone marrow into circulation; such systemic shifts may indirectly influence B-cell distribution. Moreover, B-cell homing is a multistep process [59]. While glucocorticoid-induced CXCR4 upregulation appears central [60], it likely functions in concert with other receptors (e.g., CCR7) and adhesion molecules, such as VLA-4/VCAM-1 interactions [61,62,63,64]. Thus, the GR–CXCR4 axis should be viewed as a regulatory hub within a cooperative network rather than a solitary mechanism.

Regarding methodology, our reliance on young adult male C57BL/6J mice limits generalizability. Sex and age strongly affect glucocorticoid signaling, HPA axis reactivity, bone marrow niche composition, and B-cell kinetics. For example, estrogens modulate glucocorticoid receptor sensitivity and CXCL12/CXCR4 dynamics. Given that male mice were chosen to minimize estrous-related variability, our results may not extend to females or aged cohorts. Future studies should incorporate female mice across estrous phases and aged models to assess sex- and age-specific differences.

Caution is warranted when extrapolating murine data to humans. Nonhuman primates (NHPs) provide a critical bridge, as they share cortisol-based HPA axis dynamics, glucocorticoid receptor sensitivity, and CXCR4/CXCL12 signaling closely aligned with human physiology. Thus, NHP models are essential to validate whether stress- or therapy-induced B-cell recompartmentalization parallels human responses, thereby informing the safety and efficacy of glucocorticoid receptor antagonists (e.g., mifepristone/RU486) or CXCR4 blockers (e.g., AMD3100) before clinical trials.

Beyond classical neuroendocrine pathways, systemic shifts in humoral proteins and macromolecular concentrations may also influence B-cell dynamics, representing an area for further study. Our earlier work showed that environmental cold activates TRPM8-dependent renin–angiotensin–aldosterone signaling, producing acute hypertension and vascular semipermeability that concentrates circulating proteins, including immunoglobulin G (IgG), and thereby inducing an intravenous immunoglobulin (IVIg)-like immunosuppressive state [4]. Given that B cells generate and respond to humoral immunity, an elevated endogenous IgG—or exogenous IVIg—level modulating CXCR4 expression and altering B-cell redistribution into the bone marrow remains an intriguing possibility. Future investigations into how vascular IgG shifts intersect with receptor-mediated B-cell homing will help clarify the integrative neuroendocrine–immune mechanisms underlying cold adaptation and IVIg action.

Finally, while systemic corticosterone elevation is correlated with CXCR4 upregulation in mice, direct clinical relevance remains unproven. The proposed energy-conservation model of B-cell sequestration lacks metabolic validation; future work using flux analysis is needed. The long-term consequences of modulating the CXCR4/CXCL12 axis on other hematopoietic lineages also remain unknown. Rigorous safety assessments and human studies are required before translating these acute stress insights into therapeutic strategies.

## 4. Materials and Methods

### 4.1. Animals and Ethical Approval

This study utilized adult male C57BL/6J mice (8–12 weeks old) as the primary experimental subjects. For adoptive transfer experiments, mice carrying a red-fluorescent transgene—specifically the B6.129-(Cg)-Gt(ROSA)26Sor^tm4(ACTB-tdTomato,-EGFP)Luo/JNarl strain—were selected as donors to facilitate the tracking of B-cell migration pathways under stress-induced conditions (e.g., cold exposure at 4 °C or dexamethasone treatments for 12 h). All mice were housed in a standard laboratory environment maintained at a constant temperature of 25 °C with a 12 h light/dark cycle and had free access to food and water. All animal experimental procedures strictly adhered to relevant ethical guidelines and were reviewed and approved by the Animal Care and Use Committee of Tzu-Chi University, Taiwan (Approval No. 112031).

### 4.2. Stress Induction and Pharmacological Treatments 

Ambient cold stress was induced by exposing 8- to 12-week-old C57BL/6J male mice to a temperature-controlled environment maintained at 4 °C for 12 h. During the exposure period, the mice were housed two per cage to ensure standardized environmental interaction while limiting excessive huddling. Control groups were maintained under identical conditions at a constant room temperature of 25 °C. The mice received a single intraperitoneal (i.p.) injection of 2 mg/kg dexamethasone (following previously reported dosage) [4]) to pharmacologically simulate stress-induced glucocorticoid signaling. Control animals were administered an equivalent volume of sterile normal saline as a vehicle. Following the injection, the mice were maintained at 25 °C for a 12 h observation period prior to tissue collection and analysis.

For the glucocorticoid receptor blockade, mifepristone (RU486; Sigma–Aldrich Chemie GmbH, Steinheim, Germany) was first dissolved in 100% dimethyl sulfoxide (DMSO) and subsequently diluted in sterile corn oil to a final concentration of 10% DMSO. Mice were intraperitoneally (i.p.) administered RU486 at a dose of 20 mg/kg 60 min (1 h) prior to placement in the cold chamber (4 °C). Control mice received an equivalent volume of 10% DMSO in corn oil as the vehicle.

A pharmacological blockade was executed using the specific CXCR4 antagonist AMD3100 (Plerixafor; MedChemExpress, Monmouth Junction, NJ, USA) to investigate the functional necessity of the CXCR4 signaling axis in stress-induced B-cell redistribution. Labeled leukocytes were first adoptively transferred into recipient mice and allowed to circulate for 30 min. The mice were administered an i.p. injection of AMD3100 at a dosage of 5 mg/kg or an equal volume of normal saline and then subjected to either the 4 °C cold exposure or 25 °C control environment for 12 h. All experimental and control groups were processed simultaneously to ensure consistency in downstream flow cytometric and physiological measurements.

### 4.3. Adoptive Transfer Experiments 

Donor leukocytes were isolated from the peripheral blood of red-fluorescent transgene-expressing donor mice (B6.129-(Cg)-Gt(ROSA)26Sor^tm4(ACTB-tdTomato,-EGFP)Luo/JNarl). In brief, 3 mL of blood was mixed with 9 mL of ACD in a 15 mL tube and centrifuged at 3000 rpm (Z383K; HERMLE, Gosheim, Germany) for 5 min. The supernatant was discarded, and the pellet was resuspended in 12 mL of Tyrode solution, followed by centrifugation under the same conditions. Platelets were removed by discarding the supernatant. The pellet was then resuspended in 13 mL of ACK buffer (0.15 M NH_4_Cl, 10 mM KHCO_3_, 0.1 mM Na_2_EDTA; pH 7.2–7.4) to lyse the red blood cells and centrifuged again at 3000 rpm for 5 min. After 12 mL of supernatant was removed, the pellet was resuspended in the remaining 1 mL. From this suspension, 500 µL of the leukocyte–ACK mixture was transferred to a 1.5 mL Eppendorf tube, supplemented with 1 mL ACK buffer, and centrifuged at 3000 rpm (Heraeus Biofuge Pico; Thermo Fisher Scientific, Waltham, MA, USA) for 5 min. The supernatant was discarded, and the pellet was resuspended in 350 µL of Tyrode solution. Leukocyte counts were determined using a hematology analyzer (KX-21N; Sysmex Corp., Kobe, Japan). Approximately 5 × 10^6^ cells were intravenously injected into the tail vein of recipient C57BL/6J mice. Following injection, a 30 min equilibration period was observed to allow the systemic circulation of transferred cells before subjecting the mice to stress stimuli, including 4 °C cold exposure or dexamethasone treatment.

At 12 h post-treatment, the mice were euthanized for comprehensive tissue collection. Peripheral blood was harvested via orbital sinus into EDTA-treated tubes. Spleens were manually isolated and processed into single-cell suspensions. Bone marrow cells were harvested by flushing the medullary cavities of intact femurs and tibias using PBS supplemented with 2% fetal bovine serum. All collected tissues were filtered to remove debris and immediately processed for flow-cytometric analysis to identify and quantify the redistribution of the red-fluorescent donor populations within the recipient compartments.

### 4.4. Analyses of Leukocyte Population

Single-cell suspensions were prepared from peripheral blood, spleens, and bone marrow to evaluate the redistribution and physiological state of immune populations. Erythrocytes were eliminated using ACK lysis buffer (0.15 M NH_4_Cl, 10 mM KHCO_3_, 0.1 mM Na_2_EDTA; pH 7.2–7.4), followed by washing and resuspension in FACS buffer (PBS supplemented with 2% fetal bovine serum). Cell viability was assessed using the zombie NIR kit (BioLegend, San Diego, CA, USA), an amine-reactive fluorescent dye that detects cells with compromised membrane integrity.

For immunophenotyping, the leukocytes were stained with lineage-specific monoclonal antibodies: anti-CD3 (clone 17A2, BioLegend, San Diego, CA, USA) for T cells, anti-CD45R (also known as B220; clone RA3-6B2, Abcam, Cambridge, UK) for B cells, and anti-Ly6C (clone AL-21, BD Biosciences, San Jose, CA, USA) for monocytes. The antibodies were diluted appropriately (CD3: 1000×, CD45R: 3000×, Ly6C: 5000×) and incubated with cells in Tyrode solution (50 µL) for 30–60 min. After centrifugation at 3000 rpm (Heraeus Biofuge Pico, Thermo Fisher Scientific, Waltham, MA, USA) for 5 min, the supernatants were discarded, and the cells were resuspended in PBS (200 µL). For CD3 staining, biotin-conjugated primary antibodies were obtained and incubated with fluorescence-labeled streptavidin (CF^®^405S Streptavidin, Biotium, Fremont, CA, USA).

CXCR4 surface expression was analyzed using Alexa Fluor^®^ 700-conjugated anti-mouse CXCR4 antibody (Recombinant Monoclonal Rat IgG2B, clone #247506R, R&D Systems, Minneapolis, MN, USA), diluted 1000×, and incubated with cells in Tyrode solution for 30 min. After centrifugation and resuspension, the double-stained cells were analyzed. Data were acquired using a FACS LSR II flow cytometer (BD Biosciences, San Jose, CA, USA) and subsequently analyzed with FlowJo software (version 10.8.1, TreeStar Inc., Ashland, OR, USA). The results are expressed as either mean fluorescence intensity or the percentage of positive cells within each leukocyte subset.

For accuracy and bias reduction, population-conforming elliptical and polygonal gates were applied to capture natural cell cluster contours. The gating hierarchy proceeded as follows: (i) FSC versus SSC to isolate lymphocyte/mononuclear cells, (ii) identification of distinct subsets using specific surface markers (B cells, T cells, monocytes), and (iii) quantification of CXCR4 expression within these defined boundaries.

In the adoptive-transfer experiments with tdTomato^+^ donor leukocytes (Figure 4, Figure 5 and Figure 7), recipient single-cell suspensions were gated hierarchically to identify and quantify homed donor populations while strictly eliminating nonspecific background noise. A minimum of 100,000 total events per bone marrow and spleen sample (and 50,000 total events per blood sample) were acquired across all biological replicates to standardize analysis. Initially, the mononuclear leukocyte population was selected using FSC vs. SSC. Within Gate A, the adoptively transferred donor-derived cells were identified and isolated by gating on SSC vs. red fluorescence (Gate D, e.g., Figure 4(B5), Figure 5(A2) and Figure 7(B2)). Nonfluorescent control groups (recipient mice transferred with B cells without fluorescence; Figure 4(B1–B3,G1–G3)) were utilized as reference gating templates to account for baseline background events. In these control profiles, the red color represents cell populations with low red fluorescence (basal level), and the blue color represents populations with relatively high red fluorescence caused by physical properties (such as large cell size or intrinsic tissue autofluorescence). Given that this baseline upper-left background was virtually identical across control and experimental panels (e.g., comparing Figure 4(B2,B5,B8) in the bone marrow), Gate D was positioned immediately above this nonspecific background to exclude it. Only the “extra population” shifting into Gate D in the treated groups Figure 4(B4–B9,G4–G9) was captured as true homed donor cells. Within this gated red-fluorescent donor compartment (Gate D), downstream histogram analysis (Gate J) was customized for distinct experimental purposes: For the lineage-specific identification in Figure 5, cell lineage markers CD3, CD45R, and Ly6C were analyzed (Gate J) to define and quantify the percentages of homed donor T cells, B cells, and monocytes, respectively (e.g., CD3 in Figure 5(A3); CD45R in Figure 5(D3); Ly6C in Figure 5(G3)). For the functional homing and blockade analysis in Figure 7, CXCR4 expression levels on the transferred donor cells were analyzed (Gate J, e.g., Figure 7(B3)) to evaluate the receptor-mediated targeting and the blocking efficacy of the CXCR4 antagonist AMD3100 (Figure 7(D1–G3)). This integrated background-exclusion and hierarchical gating strategy ensured high specificity and eliminated false-positive autofluorescent signals across all adoptive transfer assays.

### 4.5. Physiological and Humoral Measurements

Systemic physiological changes were monitored by measuring total leukocyte counts in the peripheral blood using a KX-21N hematology analyzer (Sysmex Corp., Kobe, Japan). For the assessment of lymphoid organ responses, spleen weight was recorded upon harvest, and its percentage was calculated relative to the total body weight of each individual mouse. Humoral signals were quantified from plasma samples via Enzyme-Linked Immunosorbent Assay (ELISA). Systemic corticosterone levels were measured using an ELISA kit (Cayman Chemical, Ann Arbor, MI, USA) in strict accordance with the manufacturers’ standardized protocols to confirm HPA axis activation.

### 4.6. Statistical Analyses

For all statistical analyses in this study, the sample size n refers strictly to the number of individual mice (independent biological replicates) in each experimental group. No technical replicates (multiple measurements from the same biological sample) were treated as independent statistical units or pooled to artificially inflate the sample size. In cases where technical replicates were performed for flow cytometry acquisitions, the technical values were averaged to generate a single representative value for that individual animal (*n* = 1). All quantitative data are expressed as mean ± standard deviation (SD). For all analyses, each “n” represents a single, independent biological replicate (one individual mouse). No technical replicates were treated as independent statistical units. Data from independent experimental runs utilizing identical protocols were pooled to achieve the final sample sizes indicated in the figures.

Statistical assumptions of normality and homogeneity of variance were verified prior to parametric hypothesis testing. Normality was evaluated using the Shapiro–Wilk normality test, and variance equality was assessed using the F-test (for two groups) or Brown–Forsythe test (for three or more groups). All datasets satisfied the criteria for parametric statistics.

For comparisons between two independent groups, a two-tailed, unpaired Student’s *t*-test was performed. For experiments involving multiple biological groups and multiple endpoints (three or more groups), data were analyzed using one-way ANOVA followed by Bonferroni’s post hoc test for multiple pairwise comparisons to control the family-wise type I error rate. All datasets are visualized as bar graphs superimposed with individual biological data points (dots), where each dot represents an individual mouse, to maximize data transparency. Statistical significance was defined as *p* < 0.05, with levels indicated as *p* < 0.05 (*) or *p* < 0.01 (**). All statistical calculations and graphing were performed using GraphPad Prism (version 9.0 or higher) and Microsoft Excel.

## Data Availability

The datasets utilized and analyzed in this study can be obtained and are available from the corresponding author upon reasonable request.

## Supplementary Materials

The following supporting information can be downloaded at: https://www.mdpi.com/article/10.3390/ijms27188214/s1.
