# Supplementary material for: Ambient Cold Exposure Modulates B-Cell Distribution via Glucocorticoid-CXCR4 Signaling
Source: Int J Mol Sci. 2026 Sep 15;27(18):8214. doi: 10.3390/ijms27188214 (PMC13607329; doi:10.3390/ijms27188214)
Supplement: Supplementary file 1 [file ijms-27-08214-s001.zip › ijms-4516280-supplementary.pdf]

Supplementary Materials

Ambient Cold Stress Rewires B Cell Distribution via Hormonal Signal

Hsuan-Yun Lee [1], Te-Sheng Lien[1], Der-Shan Sun [1], Guan-Ling Lin [1], Ching-Feng Cheng [2,3], Ko-Nien Shih [4], and Hsin-Hou Chang[1] *

Supplementory figure S1

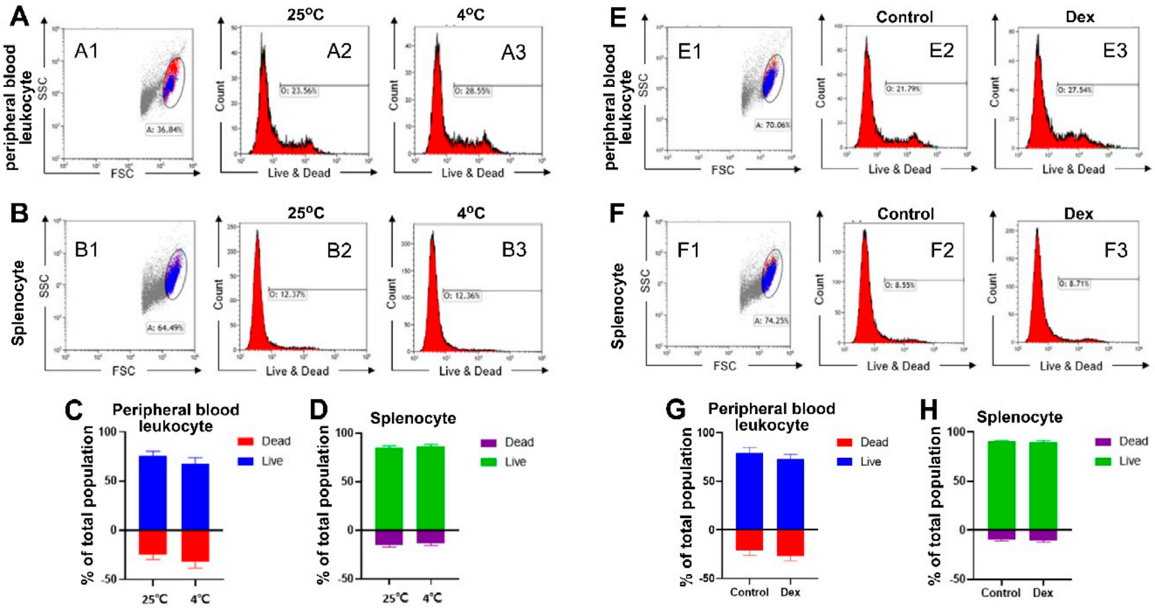

Figure S1. Cold exposure, and dexamethasone injections did not induce cell death of leukocytes and splenocytes in mice. (A–D) Results comparing cold exposure (4°C) versus room temperature (25°C). (E–H) Results comparing vehicle control versus dexamethasone (Dex) treatments. (A, E) Identification of the leukocyte population from isolated cells using forward scatter (FSC) and side scatter (SSC) (A1, E1), followed by viability analysis using the Zombie NIR kit; the percentage of fluorescent shifts represents dead leukocytes (A2, A3, E2, E3). (B, F) Identification of the splenocyte population (B1, F1) and subsequent cell death analysis using the same Zombie NIR method; fluorescent shifts indicate dead splenocytes (B2, B3, F2, F3). (C, G) Quantitative comparison of the live (blue bar) and dead (red bar) percentage of peripheral blood leukocytes 12 hours after treatments, live and dead fractions are shown as percentages of total cells. (D, H) Quantitative comparison of the living (green bar) and dead (purple bar) percentage of splenocytes 12 hours after treatments, live and dead fractions are shown as percentages of total cells. All data are presented as the mean ± standard deviation from three independent experiments. Data were collected from three experiments using 1–2 mice per group (n = 4).
